# Poly (ADP-ribose) polymerase 3 (PARP3), a potential repressor of telomerase activity

**DOI:** 10.1186/1756-9966-33-19

**Published:** 2014-02-15

**Authors:** Tamara Fernández-Marcelo, Cristina Frías, Irene Pascua, Carmen de Juan, Jacqueline Head, Ana Gómez, Florentino Hernando, Jose-Ramon Jarabo, Eduardo Díaz-Rubio, Antonio-Jose Torres, Michèle Rouleau, Manuel Benito, Pilar Iniesta

**Affiliations:** 1 Departamento de Bioquímica y Biología Molecular II, Facultad de Farmacia, Universidad Complutense y Servicios de, Instituto de Investigación Sanitaria del Hospital Clínico San Carlos (IdISSC), Madrid 28040, Spain; 2 Cirugía Torácica, Instituto de Investigación Sanitaria del Hospital Clínico San Carlos (IdISSC), Madrid 28040, Spain; 3 Oncología, Hospital Clínico San Carlos, Instituto de Investigación Sanitaria del Hospital Clínico San Carlos (IdISSC), Madrid 28040, Spain; 4 Cirugía General y del Aparato Digestivo y, Instituto de Investigación Sanitaria del Hospital Clínico San Carlos (IdISSC), Madrid 28040, Spain; 5 Centre de recherche du CHUQ – Pavillon CHUL – Cancer Axis, and Department of Molecular Biology, Cellular Biochemistry and Pathology, Faculty of Medicine, Laval University, Québec, QC, Canada

**Keywords:** PARP3, Telomerase, Lung cancer, Tumour cell lines

## Abstract

**Background:**

Considering previous result in Non-Small Cell Lung Cancer (NSCLC), we investigated in human cancer cells the role of PARP3 in the regulation of telomerase activity.

**Methods:**

We selected A549 (lung adenocarcinoma cell line) and Saos-2 (osteosarcoma cell line), with high and low telomerase activity levels, respectively. The first one was transfected using a plasmid construction containing a *PARP3* sequence, whereas the Saos-2 cells were submitted to shRNA transfection to get *PARP3* depletion. *PARP3* expression on both cell systems was evaluated by real-time quantitative PCR and PARP3 protein levels, by Western-blot. Telomerase activity was determined by TRAP assay.

**Results:**

In A549 cells, after *PARP3* transient transfection, data obtained indicated that twenty-four hours after transfection, up to 100-fold increased gene expression levels were found in the transfected cells with pcDNA/GW-53/*PARP3* in comparison to transfected cells with the empty vector. Moreover, 48 hours post-transfection, telomerase activity decreased around 33%, and around 27%, 96 hours post-transfection. Telomerase activity average ratio was 0.67 ± 0.05, and 0.73 ± 0.06, respectively, with significant differences. In Saos-2 cells, after shRNA-mediated *PARP3* silencing, a 2.3-fold increase in telomerase activity was detected in relation to the control.

**Conclusion:**

Our data indicated that, at least in some cancer cells, repression of *PARP3* could be responsible for an increased telomerase activity, this fact contributing to telomere maintenance and, therefore, avoiding genome instability.

## Background

Poly (ADP-ribose) polymerase 3 (PARP3) is a novel member of the PARP family, a group of enzymes that synthesize poly (ADP-ribose) on themselves or other acceptor proteins. Recent findings suggest that PARP3 catalyses a post-translational modification of proteins involved in biological processes, such as transcriptional regulation, energy metabolism and cell death [1,2]. Other members from this protein group, PARP1 and PARP2, have been described as active players of the base excision repair process, thus defining its key role in genome surveillance and protection [3].

Human PARP3 has been found to associate with Polycomb group proteins involved in transcriptional silencing and with DNA repair networks, including base excision repair/single-strand break repair (BER/SSBR) and nonhomologous end-joining (NHEJ), suggesting an active role for PARP3 in the maintenance of genomic integrity [3]. PARP3 has been described as a critical player in the stabilization of the mitotic spindle and in telomere integrity notably by associating and regulating the mitotic components NuMA and Tankyrase 1. Both functions open stimulating prospects for specifically targeting PARP3 in cancer therapy [4]. These findings reveal PARP3 as a positive regulator of the mitotic network containing Tankyrase 1 and NuMA with fundamental implications in spindle dynamics and telomere integrity during mitosis. Additional studies are required to determine the specific inducers of PARP3 activity [5].

As it is well known, telomere function and DNA damage response pathways are frequently inactivated in cancer. Previous results from our group indicated that telomere attrition was significantly associated with poor clinical evolution of patients affected by Non-Small Cell Lung Cancer (NSCLC), independently of tumour TNM stage. In addition, a number of genes related to DNA-repair were found significantly down-regulated in non-small cell lung tumours showing positive telomerase activity, being *PARP3* one of these molecules [6]. These data may be considered of interest in NSCLC, since *PARP3* maps in chromosome 3p (3p21.31-p21.1), and 3p deletions constitute one of the most frequent events described in relation to NSCLC. Moreover, previous results from our group and others [7] suggested the existence on 3p of one or several genes implicated on telomerase negative regulation.

Thus, considering PARP3 implication in the maintenance of genomic integrity, as well as previous results suggesting a negative correlation between *PARP3* expression and telomerase activity in non-small cell lung tumours, our main aim in this work consists of investigating in human cancer cell lines the possible role of PARP3 on the regulation of telomerase activity, which may be of relevance in the pathogenesis of NSCLC.

## Materials and methods

In order to investigate the possible role of PARP3 on telomerase regulation, we selected two human cell lines showing significantly different levels of telomerase activity. Thus, we performed “in vitro” assays on the human lung carcinoma cell line A549, with high telomerase activity, and Saos-2 human osteosarcoma cells, underlying low telomerase activity levels. The first one of the two cell systems was transfected using a plasmid construction containing a *PARP3* sequence, whereas the Saos-2 cells were submitted to shRNA transfection in order to get *PARP3* depletion.

### Cell cultures

The human lung carcinoma cell line A549 (kind gift from Dr. Paloma Navarro, Spanish National Cancer Research Centre, CNIO) was grown in Ham’s F-12 K (Kaighn’s) medium (Life Technologies, Carlsbad, California), supplemented with 10% fetal bovine serum (FBS) (Life Technologies), antibiotic-antimycotic 1X (Life Technologies) and Plasmocin™ 5 μg/ml (InvivoGen, San Diego, California). Saos-2 human osteosarcoma cells were purchased from the American Type Culture Collection (ATCC, Manassas, VA, USA), cultured in Dulbecco’s modified Eagle’s medium (DMEM, Life Technologies) and supplemented as Ham’s F-12 K (Kaighn’s) medium. Cell cultures were maintained at 37°C under 5% CO_2_.

### Plasmid transfection

A549 cells were transiently transfected with 4 μg of plasmid DNA/dish (60x15 mm) using Lipofectamine™ 2000 Reagent (Life Technologies), according to the manufacturer’s standard protocol. Plasmids used were pcDNA/GW-53/*PARP3* (containing the *PARP3* sequence of short isoform) and pcDNA-DEST53 empty vector, as control. Both were developed in our laboratory using the Gateway® (Life Technologies) Technology.

### shRNA transfection

We used the shRNA technology (SureSilencing™ shRNA Plasmids, SABiosciences, Valencia, California) in Saos-2 cells to generate stable transfectants depleted in PARP3. Four shRNAs targeting the gene of interest were supplied. As transfection system we employed magnet assisted Transfection (MATra)® (BioTAGnology, St. Louis, MO) in combination with cationic liposomes, and transfected cells with a non-functional shRNA as control. Transfected cells were selected by adding 1 μg/ml of puromycin for 3 weeks.

### RNA extraction, reverse transcription and real-time quantitative PCR (qRT-PCR)

Total RNA was extracted from A549 and Saos-2 human cells using TRIzol™ Reagent (Life Technologies) according to the manufacturer’s instructions. Reverse transcription reactions were performed with 2 μg of total RNA using the High Capacity cDNA reverse transcription kit (Applied Biosystems, USA) following the manufacturer’s instructions. Overexpression and silence of *PARP3* were determined by qRT-PCR using the Taqman probe Hs00193946_m1 (FAM™ dye-labeled TaqMan® MGB probes, Applied Biosystems).

In A549 cells, we determined the expression level of *PARP3* in transfected cells with pcDNA/GW-53/*PARP3* and pcDNA-DEST53 empty control vector, 24, 48 and 96 hours post-transfection. For quantification of gene expression, the target gene values were normalized to the expression of the endogenous reference *PPIA* (Cyclophilin A expression, Hs99999904_m1). In Saos-2 cells, *PARP3* expression level was evaluated by qRT-PCR in silenced with shRNA cells and in the transfected with the control plasmid, determining the genetic silencing ratio. The target gene values were normalized to the expression of the endogenous reference *GAPDH* (Glyceradehyde-3-phosphate dehydrogenase, Hs99999905_m1). The comparative threshold cycle (Ct) method was used to calculate the relative expression. For quantification of gene expression, the target gene value was normalized to the expression of the endogenous reference. The amount of target, normalized to the endogenous reference and relative to the control is given by 2^-ΔΔCt^ (Relative Quantification, RQ). (ΔCt = Ct _target gene_ – Ct _endogenous reference_; ΔΔCt = ΔCt _transfected_ – ΔCt _control_).

### Western-blot analysis

Fifteen micrograms of total protein were loaded on 8% SDS-PAGE and transferred to a nitrocellulose membrane (Whatman GmbH, Dassel, Germany). Blots were blocked with PBS containing 0.1% Tween-20 (PBST) and 5% powdered skim milk (PBSTM) 1 hour at room temperature and incubated overnight 4°C with rabbit polyclonal PARP3 antibody diluted 1:1000 in PBSTM (Alexis Biochemicals, San Diego, California; kind gift from Dr. Michèle Rouleau, Guy Poirier Laboratory, Québec, Canada). After washing with PBST, blots were incubated for 1 hour at room temperature with the secondary anti-rabbit antibody (Sigma-Aldrich, St Louis, Missouri) diluted at 1:1000 in PBSTM. After washing with PBST, blots were developed using Pierce ECL 2 Western Blotting Substrate (Thermo Scientific, Waltham, Massachussets). β-actin was used as loading control. Cells that expressed at higher levels the short isoform (SK-N-SH), as verified by siRNA knock down, were used as reference (kind gift from Dr. Michèle Rouleau, Guy Poirier Laboratory, Québec, Canada) [8]. Intensity of individual bands was quantified using Image J densitometry software, and expressed relative to β-actin signal, as a measure of protein relative abundance in the different conditions.

### Telomerase activity assay

Telomerase activity was determined in A549 transfected cells (24, 48 and 96 hours post-transfection) and in Saos-2 cells with the highest ratio of genetic silencing, by TeloTAGGG Telomerase PCR ELISA (Roche Applied Science, Penzberg, Germany) as previously published [9]. This method is an extension of the original Telomeric Repeat Amplification Protocol (TRAP) [10]. Briefly, in a first step, a volume of cell extract containing 10 μg of total proteins was incubated with a biotin-labelled synthetic telomerase-specific primer, and under established conditions, telomerase present in cellular extracts adds telomeric repeats (TTAGGG) to the 3′ end of the primer. In a second step, these elongation products were amplified by PCR using specific primers. An aliquot of the PCR products was denatured, hybridized to a digoxigenin labelled, telomeric repeat-specific probe, and bound to a streptavidin-coated microtiter plate. The immobilized PCR products were then detected with an antibody against digoxigenin that was conjugated to peroxidase. Finally, the probe was visualized by virtue of peroxidase-metabolizing TMB to form a coloured reaction product and semiquantified photometrically (450 nm). Thus, considering that the cut-off for telomeric repeat amplification protocol-ELISA negativity corresponds to optical density (OD)450 nm less than 0.2, all samples with OD450nm >0.2 were considered as telomerase positive. As positive control we used an extract of the telomerase-positive embryonic kidney cell line 293, and negative controls were prepared in each case by treating cell extracts with DNase-free RNase. The sensitivity of the procedure was sufficient to detect telomerase activity in an extract that contained 10 cell of the telomerase-positive cell line used as control. To avoid the effect of Taq polymerase inhibitors present in the cell extracts, we estimated the activity of telomerase by serial dilutions of each extract as described previously [11].

Telomerase activity ratios were determined as follow: [Absorbance (450_nm_) of the protein extracts from A549 cells transfected with pcDNA/GW-53/*PARP3* vector]/[Absorbance (450_nm_) of the protein extracts from A549 cells transfected with pcDNA-DEST53]; [Absorbance (450_nm_) of the protein extracts from Saos-2 cells with the highest decrease of *PARP3*, silenced with shRNA]/[Absorbance (450_nm_) of the protein extracts from Saos-2 cells, transfected with a non-functional shRNA].

PCR products were separated by polyacrylamide gel electrophoresis (PAGE), blotted onto a positively charged membrane, and chemioluminiscent detection was performed.

### Statistical analysis

Statistical analyses were developed using IBM SPSS Statistics 19 software. The paired samples *T* test was used for comparing the means of two variables, after testing normality condition by one sample Kolmogorov Smirnov test (K-S test).

## Results

### Transient over-expression of *PARP3* and decrease in telomerase activity in A549 cell line

Initially, we evaluated mRNA *PARP3* levels by qRT-PCR in A549 cell line to provide reference values. Moreover, we checked telomerase activity in this cell line. Results revealed that the enzyme was highly active in A549 cells. Our data indicated that A549 cell line showed a Delta Ct = 8.88, according to results from qRT PCR for *PARP3* analysis. In order to validate these data, we evaluated telomerase activity and *PARP3* expression in a cell line from similar origin, such as H522 (stage 2, adenocarcinoma, non-small cell lung cancer). In this case, high levels of telomerase activity correlated with similar values to those of A549 cell line for *PARP3* expression (Delta Ct = 9.14). Thus, it was considered that the best approach was to overexpress *PARP3* in this cell line in order to check if telomerase activity decreased.

**Figure 1:**
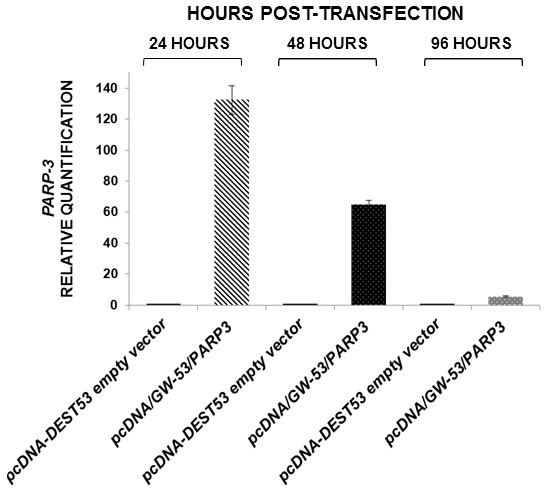
**Time course of *PARP3 *expression levels by qRT-PCR after transient transfection, in A549 cells.** Bars are the average of three experiments, media ± standard error.

After *PARP3* transient transfection, qRT-PCR was performed to measure the relative expression level of *PARP3*. Data obtained indicated that twenty-four hours after transfection, up to 100-fold increased gene expression levels were found in the transfected cells with pcDNA/GW-53/*PARP3* in comparison with the transfected cells with the empty vector. Forty-eight hours after transfection, > 60-fold increased, and 96 hours after, *PARP3* mRNA levels in the transfected cells with pcDNA/GW-53/*PARP3* were similar to *PARP3* mRNA levels in the transfected cells with the empty vector (Figure 1).

**Figure 2:**
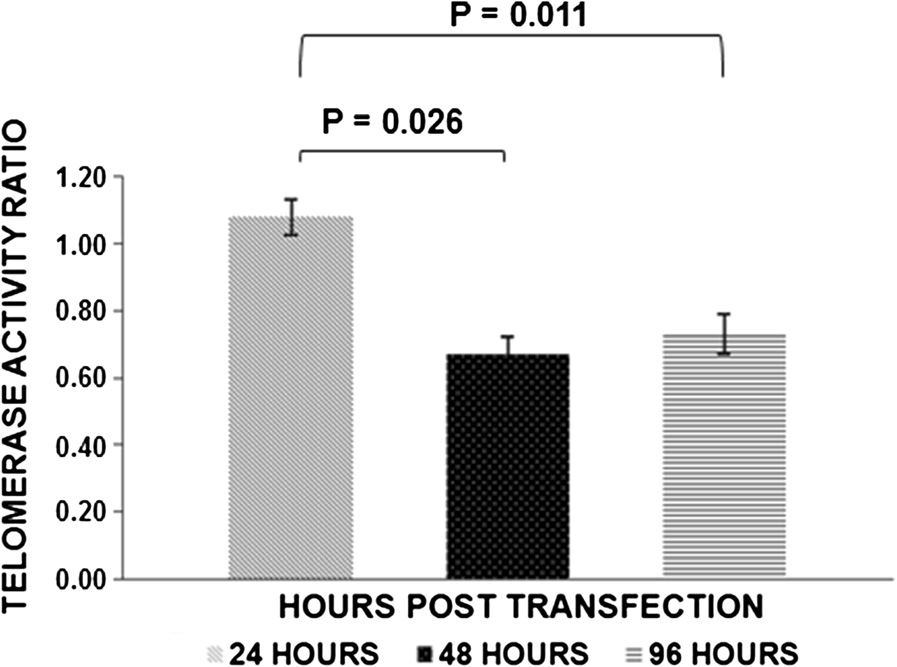
**Telomerase activity in A549 cells after transient transfection.** Time course of telomerase activity ratios [Absorbance (450 _nm_) of the protein extracts from A549 cells transfected with pcDNA/GW-53/*PARP3* vector]/[Absorbance (450 _nm_) of the protein extracts from A549 cells transfected with pcDNA-DEST53], after transient transfection. (Data are the average of four experiments, media ± standard error).

**Figure 3:**
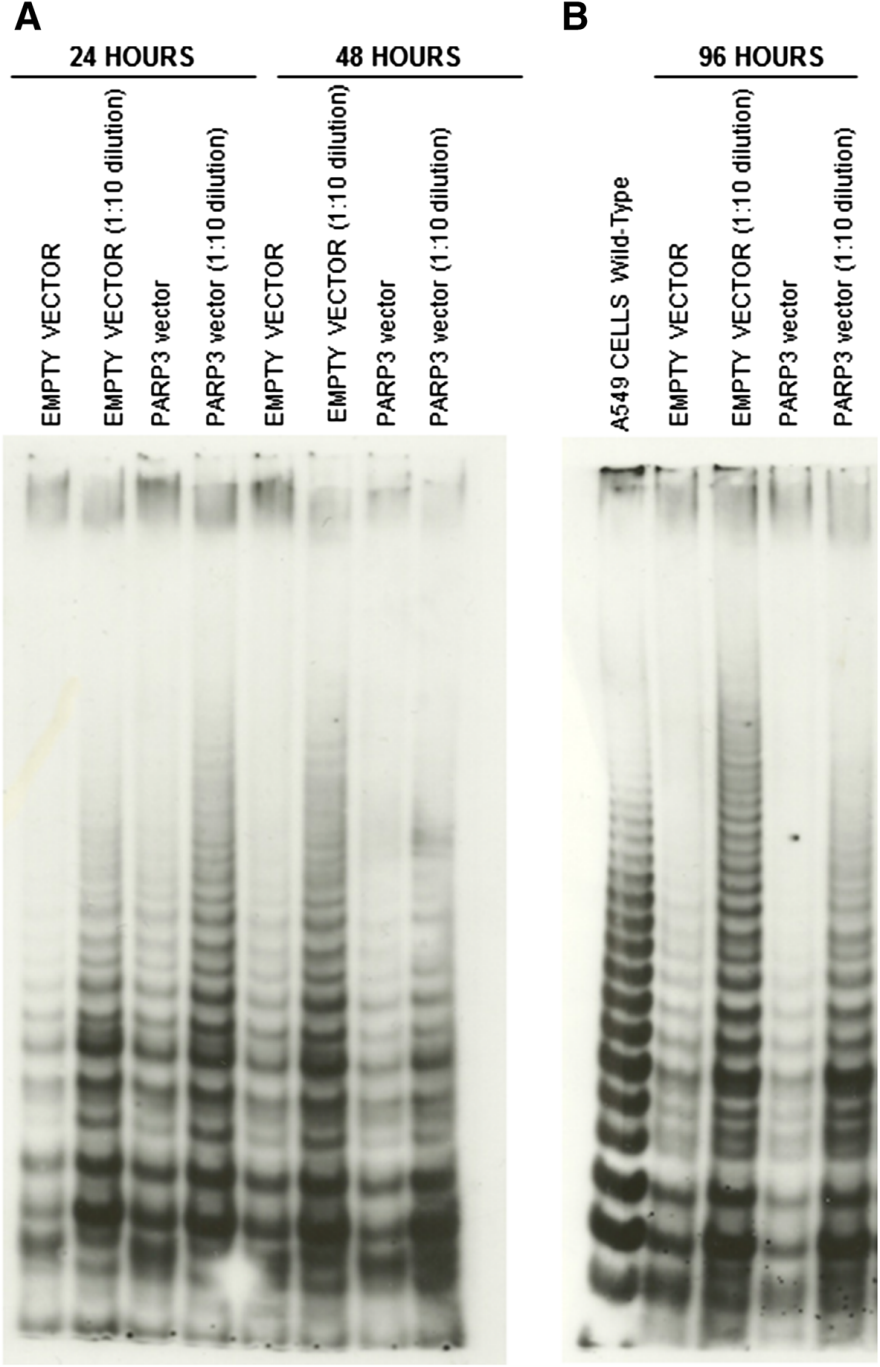
**Representative examples of telomerase activity on Polyacrylamide Gel Electrophoresis (PAGE) in A549 transfectants are shown. (A)** 24 and 48 hours after trasfection. **(B)** 96 hours after transfection.

**Figure 4:**
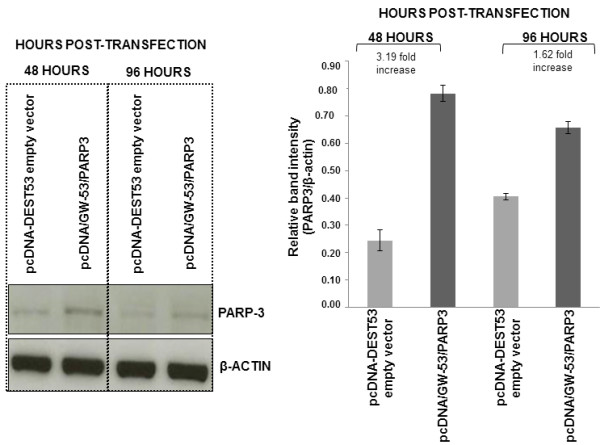
**Western-blot assay for testing PARP3 protein levels in A549 cells after transient transfection.** Bars are the average of three experiments, media ± standard error.

In relation to telomerase activity, 24 hours post-transfection no differences were found between transfected cells with pcDNA/GW-53/*PARP3* and transfected cells with the empty vector. Telomerase activity average ratio was 1.08 ± 0.05 (media ± standard error). Forty-eight hours post-transfection, telomerase activity decreased around 33% in the transfected cells with pcDNA/GW-53/*PARP3* in comparison with the transfected cells with the empty vector. Telomerase activity average ratio was 0.67 ± 0.05. Finally, at 96 hours after transfection, telomerase activity diminished around 27% in the transfected cells with pcDNA/GW-53/*PARP3* with regard to transfected cells with the empty vector. Telomerase activity average ratio was 0.73 ± 0.06. Significant differences between telomerase activity average ratio at 24 hours after transfection *vs.* 48 hours, and 24 hours *vs.* 96 hours were found (P-values: 0.026 and 0.011, respectively; Paired Samples *T* Test) (Figure 2). Representative examples of telomerase activity on PAGE are shown in Figure 3. Furthermore, Western-blot analysis revealed that PARP3 protein levels increased at 48 and 96 hours after transfection. As it can be observed in Figure 4, PARP3 increased 3.19 and 1.6-fold at 48 and 96 hours, respectively, in the transfected cells with pcDNA/GW-53/*PARP3* in comparison with the transfected cells with pcDNA-DEST53 empty vector.

### Decrease of *PARP3* and increase in telomerase activity in Saos-2 cell line

In the cell line Saos-2 we initially developed an approach similar to that described for the A549 line. Thus, in order to characterize this cell line we evaluated *PARP3* mRNA levels by qRT-PCR, and analyzed telomerase activity. Results revealed low levels of enzyme activity. Following, we performed experiments aimed at silencing *PARP3* in this cell line, then checking whether this silencing led to an increase in telomerase activity in cells.

**Figure 5:**
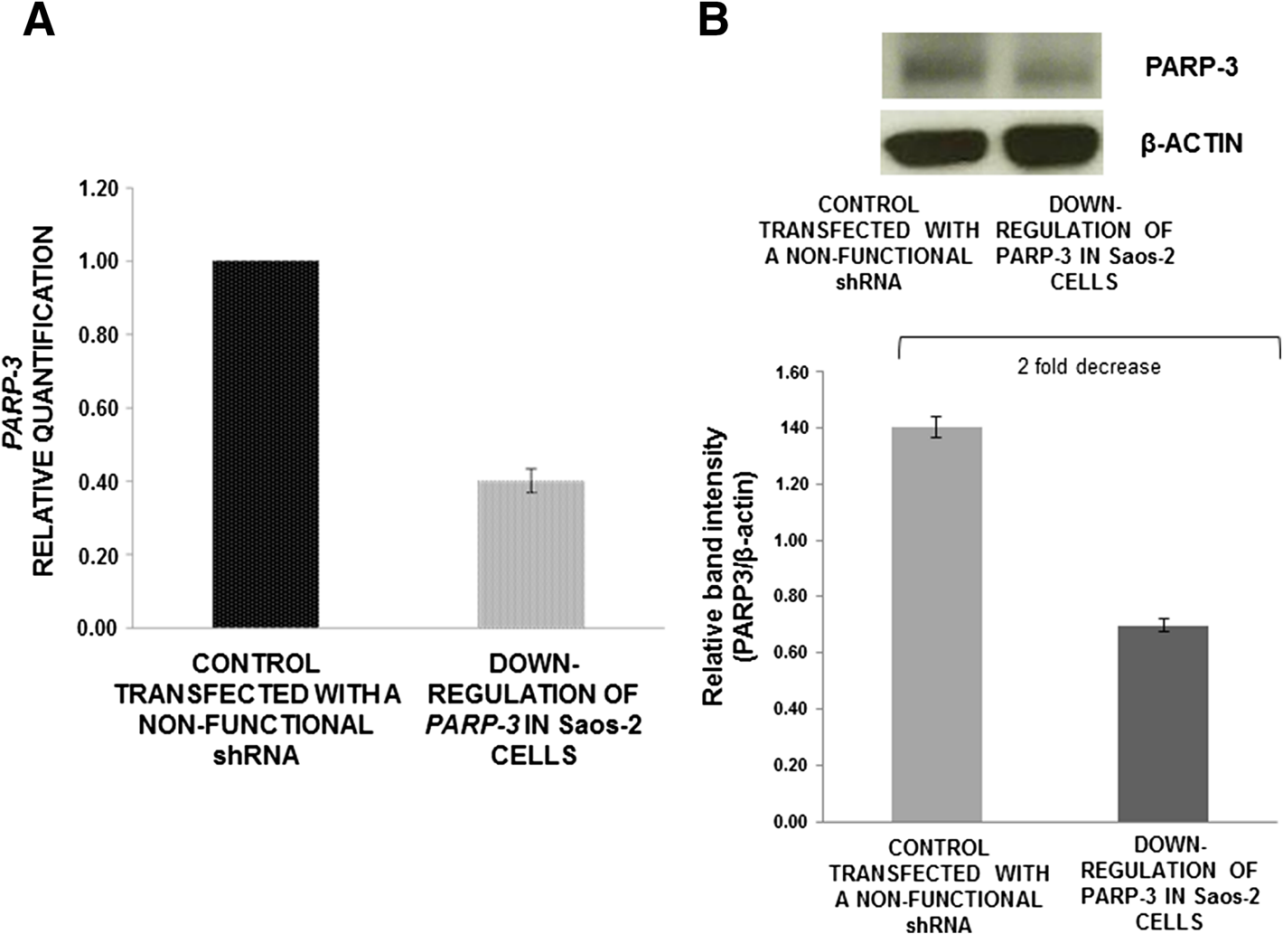
**PARP3 mRNA expression and protein levels in Saos-2 cells after transfection. (A)** Analysis of *PARP3* expression levels by qRT-PCR, after shRNA transfection (data are the average of triplicate experiments, media ± standard error). **(B)** Western-blot assay for testing PARP3 protein levels in Saos-2 cell line (bars are the average of three experiments, media ± standard error).

shRNA-mediated gene silencing allowed us to select the clone of Saos-2 cells with the highest reduction of *PARP3*, whose mRNA levels decreased by 60% with respect to the control, as qRT-PCR assays showed (Figure 5A). Western-blot was used to validate the results of quantitative real-time PCR: PARP3 protein levels decreased more than 2-fold in Saos-2 cells with the highest decrease of PARP3, compared to the control (Figure 5B).

**Figure 6:**
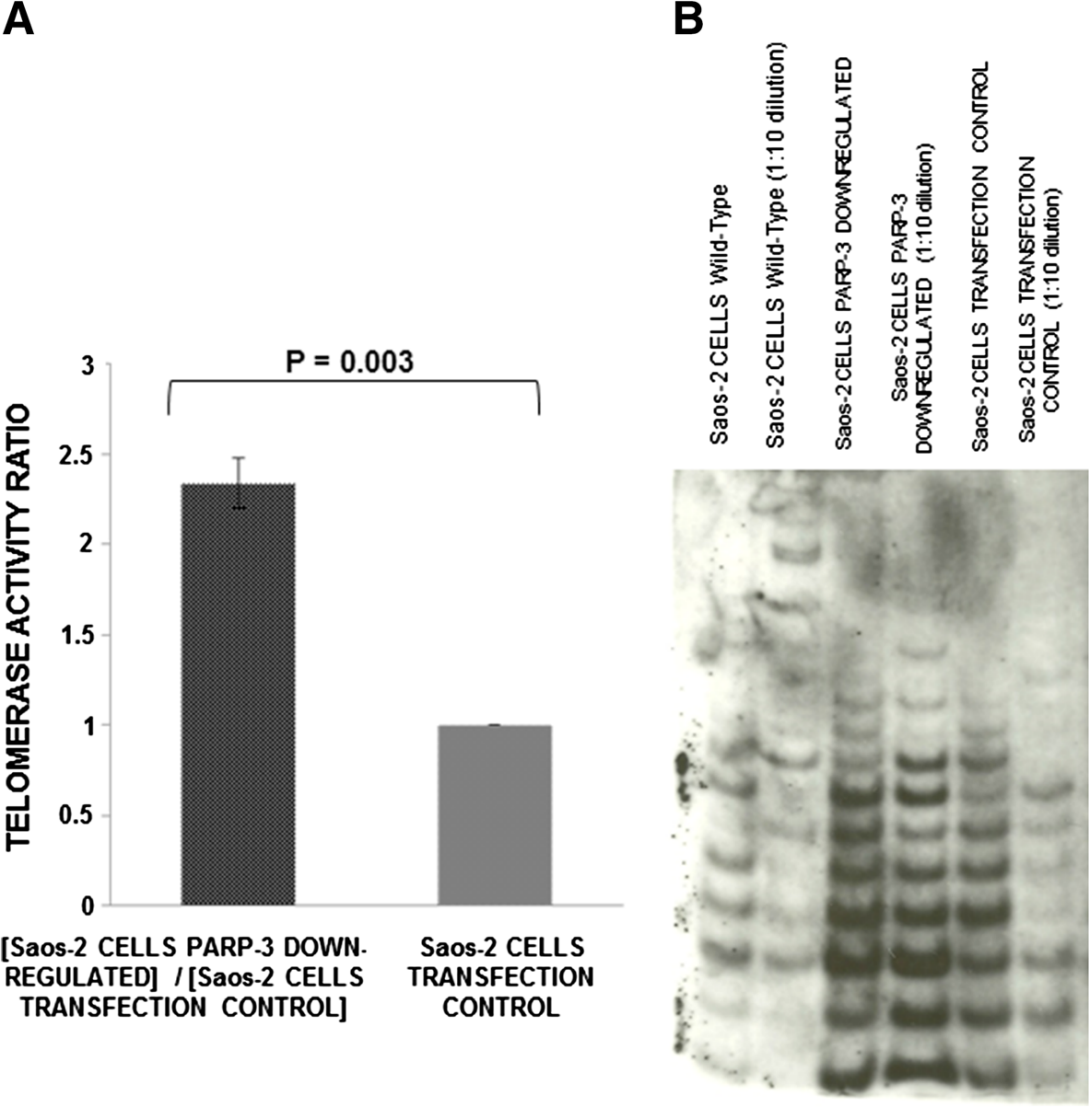
**Telomerase activity in Saos-2 cells after transfection. (A)** Telomerase activity ratios [Absorbance (450 _nm_) of the protein extracts from Saos-2 cells with PARP3 down-regulated]/[Absorbance (450 _nm_) of the protein extracts from Saos-2 cells control] (data are the average of three experiments, media ± standard error). **(B)** Telomerase activity on Polyacrylamide gel Electrophoresis (PAGE).

The clone of Saos-2 cells with the highest decrease of *PARP3* expression showed a significant (P-value: 0.003, Paired Samples *T* Test) increase in telomerase activity (2.3-fold increase), compared to the control, which was transfected with a non-functional shRNA (Figure 6A). As before, telomerase activity results on PAGE are shown (Figure 6B).

## Discussion

The considerable progress in the science of PARPs in the last years has introduced these proteins function as a key mechanism regulating in a wide variety of cellular processes including, among others, telomere homeostasis. Recently, De Vos et al. have suggested that one of the major missions for the coming years in the PARP field is to further dissect the biological activities of the emerging DNA-dependent PARPs (i.e. PARP3, Tankyrase), and to exploit their known structural features for the rational design of selective and potent PARP inhibitors [12]. Recent results identified PARP3, the third member of the PARP family, as a newcomer in DBS repair [13,14]. PARP3 has been found to regulate mitotic progression by stimulating the Tankyrase 1 catalyzed auto (ADP-ribosyl) ation and hetero (ADP-ribosyl) ation of the mitotic factor NuMA (nuclear mitotic apparatus protein 1) [14]. Tankyrase 1 is denoted as a telomere associated PARP involved in the release of the telomeric protein TRF1, via its PARsylation to control access and elongation of telomeres by telomerase [15].

In this work, we observed that *PARP3* depletion in lung cancer cells resulted in increased telomerase activity. Moreover, in cancer cells with low telomerase activity, *PARP3* showed high expression levels. These results seem to indicate an inverse correlation between telomerase activity and *PARP3* expression in cancer cells. According to our data, in A549 cells the highest mRNA *PARP3* levels were detected 24 h after transfection. However, the highest levels for depletion in telomerase activity ratio were achieved 48 h after transfection, obtaining similar values to these 96 h after transfection. This depletion in telomerase activity correlates with the highest levels in PARP3 protein. Therefore, our results seem to indicate that PARP3 could act as a negative regulator of telomerase activity. Several studies have provided insights into the biochemical and structural properties of PARP3 [13,16]. However, its physiological functions remain unknown. Recently, it has been provided evidence for two distinct roles of PARP3 in genome maintenance and mitotic progression [4]. Thus, a role of PARP3 in cellular response to DNA damage, in response to DSBs, has been emphasized. Also, it has been suggested a functional synergy of PARP1 and PARP3 in cellular response to DNA damage. Boehler et al. also discovered essential functions of PARP3 in orchestrating the progression through mitosis by at least two mechanisms, including promotion of telomere integrity [4]. We now propose a potential negative correlation between *PARP3* levels of expression and telomerase activity that also could result in telomere dysfunction. In fact, we had observed in NSCLC a significant *PARP3* down-regulation in telomerase positive tumors in relation to telomerase negative cases. Also, in NSCLC we had demonstrated a poor clinical evolution of patients affected by tumors in which telomere attrition was detected [6].

Our results suggest that the role of PARP3 in maintaining telomere integrity could be performed though regulation of telomerase activity. Therefore, depletion of *PARP3* expression could result in a defective telomerase activity. According to this hypothesis, previous experimental data had demonstrated that several normal human chromosomes, including chromosomes 3, 4, 6, 7, 10, and 17, repress telomerase activity in some cancer cells [17]. Thus, Horikawa et al. identified the E-box downstream of the transcription initiation site that was responsible for telomerase repressive mechanisms restored by normal chromosome 3 targets. This E-box-mediated repression is inactivated in various types of normal human cells and inactivated in some, but not all, hTERT-positive cancer cells. These findings provide evidence for an endogenous mechanism of hTERT transcriptional repression, which becomes inactivated during carcinogenesis [18].

In Non-Small Cell Lung tumors, we had previously described a negative correlation between *PARP3* expression and telomerase activity [6]. In fact, we detected that *PARP3* showed a significant down-regulation in association with telomerase activity. *PARP3* maps in chromosome 3p (3p21.31-p21.1), and chromosome 3p deletions constitute one of the most frequent events described in relation to NSCLC pathogenesis. Additional previous data from our group and others [7] also suggested the existence on 3p of one or several genes implicated on telomerase negative regulation.

Therefore, data reported in this work contribute to demonstrate that PARP3 could act as a negative repressor of telomerase activity with relevance in NSCLC.

PARP3 has been proposed as a new, potential target to suppress centrosome clustering, with interest in cancer therapy [4]. At this respect, our data indicate that, at least in some cancer cells, repression of *PARP3* could be responsible for an increased telomerase activity, this fact could contribute to telomere maintenance, and avoid genome instability. However, the usefulness of PARP3 inhibition in cancer therapy should also consider that repression of PARP3 could increase telomerase activity levels with a clear relation to a proliferative advantage in cancer cells.

## Conclusions

Data from this work seem to indicate that PARP3 could acts as a negative regulator of telomerase activity. *PARP3* depletion could be responsible for an increased telomerase activity; this fact could contribute to telomere maintenance, and avoid genome instability.

## Competing interests

The authors declare that they have no competing interests.

## Authors’ contributions

TFM and CF carried out most of the molecular studies, the statistical analysis, participated in interpretation of data, and were involved in drafting the manuscript. IP, CDJ and JH participated in molecular analysis and interpretation of data. AG, FH and JRJ participated in analysis and interpretation of data, as well as in advice on possible clinical implications of results from this work. MR supplied the PARP3 antibody and the SK-N-SH cells as control for Western-blot. EDR, AJT and MB have been involved in revising the manuscript. PI carried out the design and coordination of the study, and drafted the manuscript. All authors have read and approved the final manuscript.
